# Human recreational activity does not influence open cup avian nest survival in urban green spaces

**DOI:** 10.1007/s11252-024-01669-0

**Published:** 2025-01-30

**Authors:** Chloe A. Cull, Mackenzie J. Guest, Barbara Frei, Carly D. Ziter

**Affiliations:** 1 Department of Biology, Concordia University, Montreal, QC Canada; 2 Science and Technology Branch, Environment and Climate Change Canada, Montreal, QC Canada

**Keywords:** Nest survival, Urban ecology, Recreation, Urban green spaces, Avifauna

## Abstract

The breeding period of birds is a critical and sensitive portion of the annual cycle. Understanding how human use of urban green spaces affects nest survival can improve our understanding of conserving breeding bird populations in cities and support science-based management of urban green spaces that benefit both people and nature. We conducted a nest survival field study between April and August of 2023 in multiple green spaces in Montreal, Quebec, Canada, the country’s second-largest city. We asked whether human presence (distance to trails and amount of human activity) influences the nest survival of four common open-cup nesting bird species: American robins (*Turdus migratorius*), gray catbirds (*Dumetella carolinensis*), Northern cardinals (*Cardinalis cardinalis*), and yellow warblers (*Setophaga petechia*). We also asked if variables traditionally associated with nest survival, such as vegetation concealment and seasonality, would influence nest survival. Our analyses surprisingly revealed no significant influence of human activity, vegetation concealment, and seasonality on nest survival for our target species. We found for nests that did fail, nests established during the earlier part of the nesting period failed faster. American robin nests were the most successful of our study’s four target species, whereas Northern cardinal nests were the least successful. Within the limitations of our study system, our findings suggest that human presence on trails is not negatively impacting the nesting success for our target bird species using urban green spaces. Our study provides integrated science advice to land managers so they can support opportunities for people to connect with nature without causing trade-offs with biodiversity conservation.

**Supplementary Information:**

The online version contains supplementary material available at 10.1007/s11252-024-01669-0.

## Introduction

Increasing levels of urbanization have impacted biodiversity across the planet (McDonald et al. 2020), and can reduce people’s access to and connection with nature. However, natural areas in cities are increasingly recognized for their value in providing support for wildlife and services for people. Birds often take refuge in city parks and forests, hereafter referred to as “urban green spaces”, which are defined as delineated patches of land containing natural components (trees, shrubs, grasses, gardens) (De Haas et al. 2021). For example, in Montreal, researchers found a higher abundance and diversity of shrub-nesting birds in parks compared to residential areas (Rousseau et al. 2015), implying that these are important spaces for avian conservation (Buron et al. 2022). Parks also have more opportunities for coordinated efforts to improve habitat quality and diversity for birds. In contrast, residential spaces like backyards and gardens are more difficult to manage at a large scale. Individual property owners have varied preferences for vegetation and may choose ornamental, non-native plants that are generally less preferred by wildlife although their role in supporting biodiversity may be overlooked (Threlfall et al. 2017; Schlaepfer 2018). Urban green spaces provide wildlife, including birds, with shelter, areas to raise young, and food, which is particularly important in cities where green cover is low (Vasquez and Wood 2022).

In urban settings, birds are widespread, known indicators of biodiversity (Fraixedas et al. 2020), and are beneficial to humans and society (Buxton et al. 2024). Cities are often found along major migration flyways with birds using urban green spaces to rest and replenish fat stores (Rodewald and Matthews 2005; Seewagen et al. 2010).Artificial light pollution in cities can lead to aggregations of nocturnal migratory birds, as they often rely on visual cues and become confused by the bright light (Van Doren et al. 2017). During the breeding period, urban green spaces can be attractive to open cup shrub-nesting bird species specifically, as cities often have many of their preferred shrubby edge habitats (Kurucz et al. 2021). Typically, open-cup nesting species have lower rates of nest survival in comparison to species that use other nesting strategies, e.g., cavities (Ospina et al. 2018). Their nests are often and easily accessed by mesopredators, such as raccoons and squirrels, which are common in cities in the absence of sensitive apex predator species (Morosinotto et al. 2012). Cities present many general dangers to birds, including building and window collisions, and novel predators (Van Doren et al. 2021; López-Flores et al. 2009). Furthermore, the fragmented nature of urban green spaces and other habitats in cities can pose challenges to birds as it can limit their movement and increase vulnerability to predation and competition (Marzluff 2001). Indeed, a recent publication showed that most bird species cannot tolerate a high level of human pressure, with only 22% of over 6000 global species of birds being more tolerant (Marjakangas et al. 2024) – emphasizing the importance of managing green spaces to reduce urban pressures when possible.

Responsibility for protecting and managing urban green spaces is shared between federal or provincial initiatives and local municipal governance. In Canada, for example, federal initiatives such as the National Urban Parks initiative, the Growing Canada’s Community Canopies initiative, and the Nature Smart Climate Solution Fund provide funding and/or guidance to promote the conservation, restoration, and management of urban nature. (Parks Canada 2023; Natural Resources Canada 2024; Environment and Climate Change Canada 2024). The establishment, protection, and management of urban green spaces is particularly important for biodiversity in southern Canada, as this region is heavily developed, but also contains the majority of the country’s species at risk. Furthermore, southern Canada has experienced low conservation responses relative to the threats facing biodiversity in these regions (Kraus and Hebb 2020).

In addition to protecting biodiversity, urban green spaces facilitate human-nature interactions, with urban birds representing some of the most accessible wildlife to people. Interactions with nature have been shown to reduce the frequency of violence, mortality and heart rate, while improving attention span and the frequency of physical activity (Kondo et al. 2018). Additionally, human-bird interactions specifically help to reduce symptoms of anxiety, paranoia, and depression (Stobbe et al. 2022). A recent study found a positive relationship between urban bird diversity and self-reported good mental health, indicating that cities with greater biodiversity may enhance overall mental wellbeing (Buxton et al. 2024). Furthermore, another study in Europe found bird species richness to be related to life satisfaction, showing that an increase in bird diversity on human life satisfaction was comparable to a 10% increase in salary (Methorst et al. 2021). Birds provide people with important cultural services, such as promoting feelings of relaxation and a strong connection to nature through activities such as birdwatching or bird feeding (Cox and Gaston 2016). These positive interactions provide incentive to protect and maintain our urban green spaces.

While people receive mainly positive effects from birds, studies have reported that human presence in urban green spaces can impact the diversity and abundance of bird species (Bötsch et al. 2018; Jokimäki 1999; Kangas et al. 2010; Lepczyk et al. 2008; Van der Zande et al. 1984). For example, some species are highly sensitive to noise pollution present in urban green spaces, such as long-eared owls (*Asio otus*), which are limited by noise pollution despite this species’ choice habitat being provided in urban parks (Fröhlich and Ciach 2018). Additional studies have suggested that urbanization and human presence are negatively correlated with bird species richness (Peña et al. 2022; Bötsch et al. 2018). Yet, there is a lack of consensus on how more direct measurements of bird population well-being, might be impacted by human disturbance (Chamberlain et al. 2009; Vincze et al. 2017). A strong example of a direct measurement is reproductive success, as the breeding period represents a critical and vulnerable period in a bird’s life and allows for the survival of a species (Forslund and Pärt 1995).

Research in more natural areas has suggested that human presence and recreation have negative consequences on avian reproductive success, but evidence and consensus of this in urban green spaces is lacking. Human disturbance had a significantly negative effect on nest success in seabirds in a nature park in Scotland (Beale and Monaghan 2004). Another study on blue tits (*Cyanistes caeruleu*) in experimentally placed nest boxes in a forest 60 km outside of Madrid found that nestlings hatched on holidays, linked to an increase in recreation and human gatherings, were associated with impaired development (Remacha et al. 2016). Nests closer to roadways and human development are also more likely to be abandoned by the cavity-nesting American kestrel (*Falco sparverius*) (Strasser and Heath 2013). Waterfowl are reported to spend less time at their nests due to human disturbance, thereby increasing the chance of nest predation (Stien and Ims 2015). Predation is globally known to be the primary source of nest mortality (Ricklefs 1969). Mesopredator populations, including common nest predators such as crows and raccoons, often explode in urban environments, as these spaces favour conditions that increase mesopredator survival and reproduction (Prugh et al. 2009, Kovér et al. 2015, Prange and Gehrt 2004).

Conversely, one study showed that food subsidies provided by humans in urban green spaces can help relieve predation pressure on bird nests (Rodewald et al. 2011). Furthermore, a study in a suburban area of Colorado found that experimentally placed nests closer to trails incurred less predation, likely because human presence and recreation decreased known predators of shrub and low-nesting bird species (Miller and Hobbs 2000, 2002). This is known as the “human shield” hypothesis, which proposes that in regions with higher human activity, human presence acts as a buffer against the impacts of predation on prey species, effectively shielding these animals from their predators and increasing survival (Berger 2007; Gámez and Harris 2021; Pérez-González et al. 2024). Another study on Northern cardinals (*Cardinalis cardinalis*) in Ohio publicly owned parks (including some in highly urbanized areas) found that human recreation did not have an apparent effect on nest survival, and that height was the mediator of human disturbance and likelihood of flushing (when birds leave their nest in response to disturbance) (Smith-Castro and Rodewald 2010b). They believed that nests where birds flushed their nests at further distances from humans, compared to those where birds flushed when humans were closer, would experience more predation because their nests are unoccupied for longer (Smith-Castro and Rodewald 2010b). Evidence that human disturbance both does and does not impact reproductive success highlights the need for clarity on the subject, especially as cities expand and the number of people using urban green spaces increases.

The overall aim of our work is to assess whether human presence in North American urban green spaces influences the nest survival of common species of open-cup shrub nesting birds on the island of Montreal. We also sought to understand how aspects of the vegetation in the nest patch and seasonality might influence nest survival, as these are often included in traditional nest survival studies and are known to be important predictors of nest survival (Davis 2005; Ringelman 2019). For nests that failed, we also investigated whether these variables were significant predictors of the total number of active days that a nest experiences—defined as the number of days from nest inception (first day of building) to nest failure. We did this by conducting a nest survival study across three sites, measuring vegetation variables at the nest patch, quantifying human activity using anonymous cellphone data (Filazzola et al. 2022), and distances of nests to trails, and conducting Cox proportional hazards modelling.

We predict that nesting survival would rise with greater distances from trails and higher rates of human activity, given birds’ vulnerability to disturbances posed by trail users, such as loud noises and dogs (Steven et al. 2011), which could increase flushing and signal predators to the location of the nest (Martin et al. 2000) or cause parental abandonment (Beale and Monaghan 2004; Boyd, n.d.; Strasser and Heath 2013). Additionally, we predict that nests higher from the ground, that have more vegetative concealment and shrubby vegetation would increase nest survival and the number of active days a nest experiences. We anticipated this as predators common in urban forests, including cats, raccoons, and squirrels may face more difficulty finding and accessing nests (Martin 1993; Seibold et al. 2013; Ringelman and Skaggs 2019) and that nests would be more shaded from the sun, which is crucial for regulating temperatures for healthy nestling development (Corregidor-Castro and Jones 2021). We expected that nests would experience higher rates of success and more active days with the progression of time through the season, as there is more dense foliage later in the season to conceal and shade nests and a lower likelihood of cold weather events.

## Methods

### Study sites

**Fig. 1:**
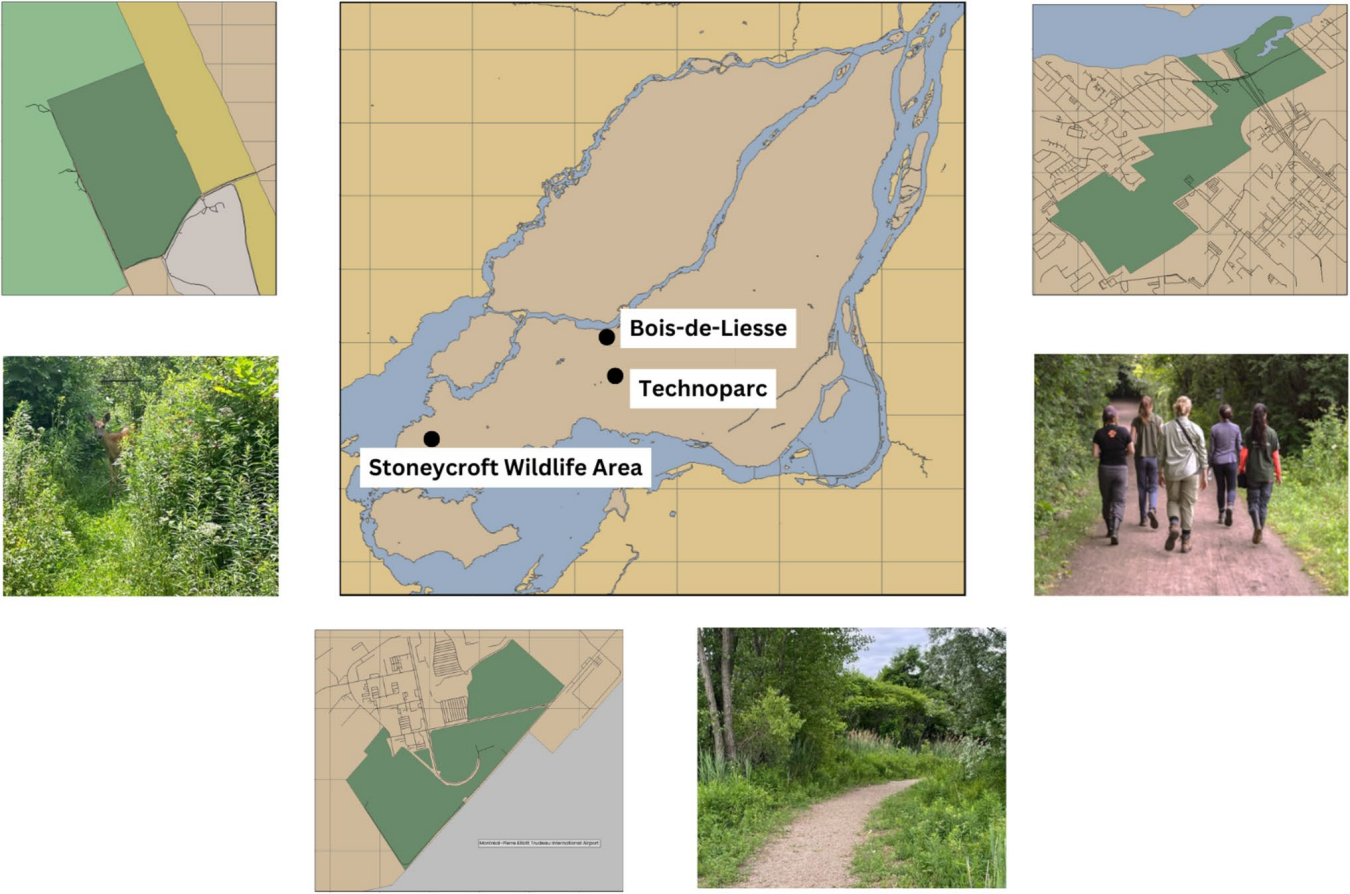
Map of the island of Montreal highlighting the three study sites included in the nest survival study. To the left of the central map, Stoneycroft Wildlife Area is shown alongside an example trail image. The Technoparc area and a corresponding trail image are positioned below the central map, while Bois-de-Liesse and an image of one of its trails are displayed to the right of the central map

The Island of Montreal is in the St. Lawrence Lowlands of Southern Quebec and contains Canada’s second largest city. The Island of Montreal covers an area of 498.29 km^2^ with a population density of 4022.3 people / km^2^ (Government of Canada 2022). The area hosts various habitats such as woodlands, wetlands, brownfields (area previously developed that has become abandoned), and shoreline habitats amongst a developed, urban landscape, allowing for approximately 145 bird species to nest each year (Québec Breeding Bird Atlas – n.d.). We conducted our study in three urban green spaces on the primarily residential and industrial west side of the Island of Montreal: Bois-de-Liesse Nature Park, Technoparc, and Stoneycroft Wildlife Area selected to span a gradient of human activity (Fig. 1). Although these sites are not in the city centre, they are still in human-dominated areas (with adjacent neighbourhoods ranging in density from approximately 500 to 4500 people per square km), and thus representative of the types of green spaces present in residential areas of many North American cities, which are characterized by relatively low-density development. While the parks differ in total size, their vegetation composition is relatively similar and all contain similar dense shrubs, which attract our target bird species. Specifically, the overall habitat composition at each of the three sites is a mix of shrubby edges and mature forest.

Bois-de-Liesse is a high visitation park managed by the city of Montreal that is used by cyclists, runners, and dog-walkers, as well as for recreational activities such as picnics and children’s day camps. Technoparc is a large green space that includes both newly acquired city land (yet to be managed for recreation) and privately owned areas. The area is informally managed by a non-profit organization and is primarily used by birders and nature photographers. Stoneycroft Wildlife Area is a private research area with no public access that is owned by McGill University and hosts a small field station that is active 6 months each year during both the migration and breeding seasons (overlapping with our sampling period by approximately 1.5 months). Our nest searching efforts in all three green spaces were focused along both small footpaths and larger, more heavily trafficked trails.

### Study species

We studied four target species that commonly nest in all our study sites: American robins (*Turdus migratorius*), gray catbirds (*Dumetella carolinensis*), Northern cardinals (*Cardinalis cardinalis*), and yellow warblers (*Setophaga petechia*). These species are generalist open cup nesters and thus have likely comparable survival and predation rates (Billerman et al. 2022; Leston and Rodewald 2006). Despite their status of “least concern”, they are still vulnerable to threats posed by human influence and a changing climate (Lindenmayer et al. 2011). Focusing on managing for common species could thus serve as a strategy to indirectly support and protect at-risk species, particularly those with similar nesting and reproductive behaviours. These common species are also more likely to be known and recognized by the general public, and are thus important species to study to better connect the results of urban biodiversity research to human wellbeing and people’s interactions with nature.

### Nest monitoring

We initiated nest searching at dawn, the period of peak avian vocalization and activity (Southworth 1969) and ended at 14:00 between April 26th and August 4th, 2023. Searching was conducted at one site per day rotating through each of the three sites. We did not search for nests on days with heavy rainfall or other inclement weather, such as thunderstorms or days with extreme heat warnings, when birds are known to be less active (Rosamond et al. 2020). Nest searching methods included both territory mapping (Smith et al. 2009) as well as opportunistic observations of target species. Behavioural cues used in finding nests included observing individuals carrying nesting material, vocalizing, or feeding young (Martin and Geupel 1993). During territorial mapping we marked the location of adult birds on a physical map each time we were in that area and approximated the extent of individual territory (Jablonski et al. 2010). We also noted the location of male birds vocalizing, as this often indicates that they were at the edge of a territory (Stoddard et al. 1991).

Once nests were found, we recorded their geographic coordinates using a Garmin GPS (Extrexx 22X). We also collected data on weather conditions, the time the nest was found, the species to which the nest belonged, the nest substrate or tree species, and the presence of potential nest predators that could be seen or heard within a 10 m radius of the nest (Purcell and Verner 1999; DeGregorio et al. 2016).

We monitored nests every three to five days, following previously established nest monitoring methods (Bailey et al. 2019). Unlike searching, we conducted nest monitoring in a variety of weather, including light rain. In some cases, nests could not be monitored by direct observation at eye level, and we used binoculars and/or an elevated endoscopic camera. We presumed the nest to be active when an adult was present incubating eggs or brooding nestlings. At each nest monitored, we collected the following data: time, date, weather, information on the status of adult birds (if present), eggs (if present), young (if present), condition of the nest (whether it was unfinished, fully constructed, flattened with fecal matter, or removed), whether predators could be observed or heard in a 10 m radius of the nest, and any other noteworthy information (Bailey et al. 2019). In some cases, we observed instances of active predation on nests, and we took note of this. If a nest had been removed from where it had once been located, we noted the condition and location of the disturbed nest.

### Vegetation surveys

We surveyed vegetation at three spatial scales as birds are known to use different variables at different spatial scales for nest site selection: the nest tree or shrub (henceforth “nest tree”), a 1 m radius nest patch, and a 5 m radius nest patch. We conducted these surveys typically within five days (but 10 surveys were conducted within 5–12 days) of the nest failure or fledging date to capture vegetation characteristics of the nest patch as accurately as possible. For each nest tree, we recorded the diameter at breast height (DBH), species, height, nest height, vegetative complexity (see Appendix B), and canopy cover above the nest using digital image analysis (see Appendix B). To estimate nest concealment, we used checkerboards (three by three alternately black-and-white squares) of three sizes to accommodate differently sized nests, adapted from Nudds’ method of estimating vegetative cover of deer (Nudds 1977; see Appendix B). For the 1 m radius vegetation survey we identified all woody stems in a 1 m radius around the nest tree to species level and tallied the number of stems based on size classes (< 1 cm, 1–3 cm, 3–6 cm, and 6–9 cm DBH). In the 5 m radius vegetation survey we identified each tree greater than 5 cm to species and measured their DBH. Lastly, we measured the distance of each nest to the nearest trail.

### Human activity indices

To investigate the human impact of nest survival, we obtained human activity indices of our three sites by using ©MapBox Movement data (www.mapbox.com/movement-data). These data were recorded from 2020 to 2022, and while they did not correspond to our study year, we assumed that the average activity in this timeframe would reflect the human activity present at our sites in 2023. Focusing on data from 2020 onwards accounts for a potential increase in outdoor activities post-covid, where many people re-discovered local green spaces and turned to outdoor recreation as a safer social activity (Geng et al. 2021). The activity indices were obtained from anonymized cellphone location data within 10 m x 10 m plots per two-hour window (following Filazzola et al. 2022). We subsequently overlaid these human activity indices with our nest GPS points through spatial analysis using R version 4.3.2 (R Core Team 2023), using the stars, sf, stringr, and dplyr packages (Pebesma and Bivand 2023; Pebesma 2018; Wickham 2023; Wickham et al. 2023) to extract mean activity indices within a 20 m radius of each nest location.

### Nest survival analysis

We used program RMark (Laake 2013) to estimate daily survival rates (DSR) of each of our target species and used the DSR to the power of the average nesting period for each species to calculate species-specific modelled nest success (Jehle et al. 2004). American robins have an average nesting period of 35 days, gray catbirds have an average nesting period of 32 days, Northern cardinals have an average nesting period of 30 days, and yellow warblers have an average nesting period of 31 days (Billerman et al., 2022). To visualize survival over time for each of our target species, we plotted Kaplan-Meier curves using ggsurvfit, ggplot2, and magrittr packages (Sjöberg et al. 2024; Wickham 2016; Bache and Wickham 2022) in R 4.3.2 (R Core Team 2023). These curves display the probability of the nest surviving each day through the nesting period.

To assess the effects of distance to trail, human activity, vegetation variables, and seasonality on nest survival, we used Cox proportional hazard modelling (Cox 1972). This modelling approach tests the effects of various covariates on the duration until nest failure while accounting for censoring, and time-varying factors (Nur et al. 2004; Roper 2003; Heisey et al. 2007; Liebezeit et al. 2009). Nest successes were our censored events, and were assigned values of “0”, whereas nest failures were assigned as “1”.

Preceding our model building, we checked our variables of interest for collinearity. We established a cutoff for collinearity of 0.7, consistent with studies in ecology (Harrison et al. 2018; Dormann et al. 2012). We calculated Pearson correlation coefficients and then visualized the correlations using the corrplot() function in R Studio and checked for any significant correlation (Wei and Simko 2021; R Core Team 2023). We also ensured that our models met the assumptions associated with Cox proportional hazards modelling. The fundamental assumption for Cox proportional hazards modelling is that the hazard of the event of interest occurring is constant over time. We assessed this assumption using the cox.zph() function from the R survival package, visually assessing plots of the residuals (Therneau 2024).

We built five models (Table 1) based on our hypotheses, aiming to identify variables (Table 2) that may impact nest survival of our target species. We included site and species as random effects variables in all models to account for the fact that our studied sampling locations were nested within sites and that the species may respond differently. Our chosen explanatory variables were regressed against the number of days the nests survived. This generated hazard ratios for nest failures over time. Hazard ratios indicate the probability that an event of interest will occur, in our case, these events were nest failures.

**Table 1:** Our built models with their explanatory variables used in Cox proportional hazards modelling of avian nest survival in Montreal urban forests. The response variable for all models was the time to nest failure. Random effects of site and species are included

Model Name	Hypothesis	Explanatories
Human Recreation Activity	The proximity of nests to trails and the average human activity around nests strongly influences nest survival.	Distance to Trail + Human Activity Indices + (1|Species) + (1|Site)
Vegetation	The vegetative cover, the height, and the density of stems surrounding nests strongly influences nest survival	Vegetation Concealment + Nest Height + # Small Stems/Ha + (1|Species) + (1|Site)
Seasonality	Seasonality, quantified by the day of year when nest building was first initiated, strongly influences nest survival	Initiation Date + (1|Species) + (1|Site)
Global		Distance to Trail (m) + Human Activity Indices + Vegetation Concealment + Nest Height + # Small Stems/Ha + Initiation Date + (1|Species) + (1|Site)
Intercept Only		(1|Species) + (1|Site)

**Table 2:** Summary statistics for variables measured and used in this study of bird nest survival (American robins, gray catbirds, Northern cardinals, and yellow warblers) in Montreal, Canada

Variable	Range	Median	Mean (+/- SD)
Distance to Trail (m)	0.10–110.00	5.20	15.63 (+/- 22.46)
Vegetative Concealment	0.00–6.00	2.19	2.22 (+/- 1.30)
Human Activity	4.16 × 10^−4^ – 0.05	4.53 × 10^−3^	0.01 (+/- 0.01)
Initiation Date (DOY)	1.00–58.00	34.50	32.17 (+/- 14.67)
Canopy Cover	0.06–45.53	1.84	6.22 (+/- 11.53)
Vegetation Density (1 m from nest)	0.00–3.18 × 10^5^	1.59 × 10^4^	2.66 × 10^4^ (+/- 4.81 × 10^4^)
Vertical Complexity	1.00–3.00	2.00	2.09 (+/- 0.71)

We fit models using the coxme() function from the Coxme (Therneau 2012) and survival (Therneau 2024) packages in RStudio 4.3.2 (R Core Team 2023). To identify the best fit model, we used the corrected version of Akaike’s information criterion (AICc), which uses a model’s maximum likelihood estimator and penalizes for the number of parameters to rank a candidate set of models (Table 3) (Lebreton et al. 1992).

**Table 3:** Daily survival rates estimated, standard errors (SE) and the percentage of successful nests for American robins (*n* = 32), gray catbirds (*n* = 11), Northern cardinals (*n* = 23), and yellow warblers (*n* = 23) using the RMARK program

Species	DSR	SE	Modelled Nest Success
American robins	0.94	0.01	11.5%
Gray catbirds	0.89	0.04	2.4%
Northern cardinals	0.82	0.05	0.03%
Yellow warblers	0.90	0.03	3.8%

### Number of active days analyses

For nests that failed, we used a multivariate linear regression model to assess the impacts of human presence variables (distance to trail, human activity index, and site), seasonality (day of year) and concealment variables (coverage, canopy cover, vertical complexity, density of surrounding vegetation). Analysis was done with packages lme4 (Bates et al. 2015) lmerTest (Kuznetsova et al. 2017), and ggplot2 (Wickham 2016). Our resulting mixed model is the following:

$$\mathrm{Active}\;\mathrm{days}\:\sim\:\mathrm{concealment}\:+\:\mathrm{canopy}\;\mathrm{cover}\:+\:\mathrm{vertical}\;\mathrm{complexity}\:+\:1\&\mathrm{nbsp};\mathrm m\;\mathrm{vegetation}\;\mathrm{density}\:+\:\mathrm{distance}\;\mathrm{to}\;\mathrm{trail}\:+\:\mathrm{human}\;\mathrm{activity}\:+\:\mathrm{day}\;\mathrm{of}\;\mathrm{year}\;\mathrm{failed}\;+\;(1\vert\mathrm{site})\;+\;(1\vert\mathrm{species})$$ 

## Results

### Nest survival of target species

We found and monitored 93 nests of our target species across three urban sites until failure or success. A nest was considered successful if at least one nestling successfully fledged. Four nests were removed from the analysis as they were determined to be outside site boundaries. An additional two nests were removed as we could not access them for proper monitoring, so their fates were unknown. This resulted in a final sample size of 87 nests (Appendix C & D). For all four target species and across all three sites, we found an overall apparent nest success rate of 24.1%. American robins had the highest modelled nest survival estimate, followed by yellow warblers, gray catbirds, and Northern cardinals had the lowest modelled nest survival estimate (Table 4).

**Table 4:** Results from candidate models predicting nest survival of American robins, gray catbirds, Northern cardinals, and yellow warblers in three urban forests of Montreal. Each model is named based on its predictors. The number of parameters (k), the corrected delta AIC (AICc), and the fitted log-likelihood are reported for each model

Model	Variables	k	∆AICc	loglik
Seasonality	Nest Initiation date	3	0	−225.86
Intercept Only	N/A	2	0.2	−226.75
Human Recreation Activity	Distance of nest from trail + Mean human activity index	4	3.6	−226.65
Vegetation	Number of small stems/hectare + nest height + vegetation concealment	5	5.3	−226.38
Global	Number of small stems/hectare + nest height + vegetation concealment + distance of nest from trail + mean human activity indices + calendar date	8	9	−225.37

**Fig. 2:**
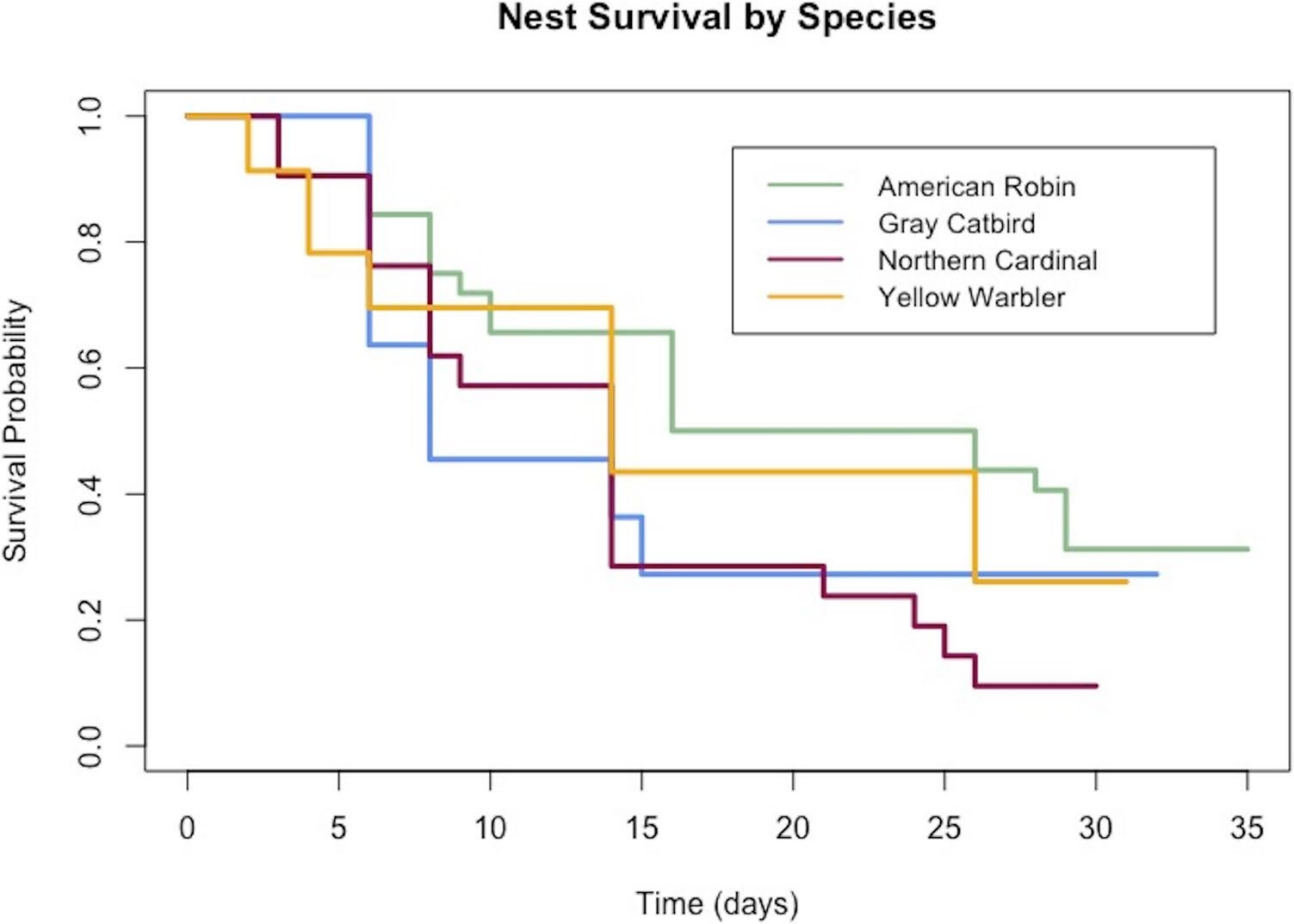
Kaplan-Meier survival curve displaying the survival probability of our four target species: American robins, gray catbirds, Northern cardinals, and yellow warblers across three urban forested sites in Montreal over time

Kaplan-Meier survival estimate curves show the probability of survival through time. We observed steep decreases in nest survival probabilities during the first half of the nesting period, and more graduate declines in the second half of the period (Fig. 2). These patterns were consistent across all four target species.

We built our Cox proportional hazard models and then ranked their fit based on AIC, providing us with measurements of how well our hypothesized models fit our nest survival data. None of our models differed enough to classify any one of them as more or less parsimonious than others (Appendix E & F).

### Determinates of number of active days in failed nests

Human presence (distance to trail) and concealment (concealment, canopy cover, vertical complexity, and vegetative density) did not have significant effects on the number of active days. These effects were similar across site (random effect variance 0.00). Seasonality (day of year) was the only explanatory variable to significantly predict the number of active days (hazard ratio: 1.01, β: −0.09, 95% CI: 0.24–0.76) prior to a nest failing, with nests that were active earlier in the season failing faster (i.e., less active days before failure).

## Discussion

We asked whether the presence of humans, nest patch vegetation, and seasonality in urban green spaces with varying degrees of human activity on the Island of Montreal predicted the nest survival of four common species of open-cup nesting passerines. For nests that failed, we explored whether these same variables predicted the number of active days nests had before failure. Surprisingly, we found that none of the chosen predictors, including human activity and distance to trails, significantly explained variations in nest success, despite varying values of the predictors across nests and sites. Although the AICc values of our models were similar to each other, seasonality was marginally better at predicting a nest’s number of active days before failure; nests active earlier in the season failed faster. These urban green spaces were thus found to accommodate human use without compromising bird success.

Studies looking at modelled nest survival of various bird species in urban landscapes have found varying results. In contrast with our results, studies focusing on more direct human impacts often find a significant influence. For example, survival in the clay-coloured thrush (*Turdus grayi*) was reduced in urban environments in Costa Rica due to the presence of brightly coloured artificial materials in their nests that attracted predators to their nests (Corrales-Moya et al. 2023). A study of shrub nesting birds in Hungary found that modelled nest survival was higher in urban areas compared to rural areas, but the researchers focused on artificially built nests (Kurucz et al. 2021). Researchers in the northeastern US investigated modelled nest survival through a lens of predation and included three of four of the target species we used: gray catbirds, Northern cardinals, and American robins. They found modelled nest survival of American robins and gray catbirds to be higher in urban areas compared to rural areas, but they did not specifically incorporate human activity in their modelling, instead looking at a gradient of urbanization (Ryder et al. 2010). Studies of COVID-19 lockdowns provide another avenue to assess the impact of human pressure on wildlife. A paper investigating the impact of COVID-19 lockdown – and an associated drastic reduction in human activity – on nesting dark-eyed juncos (*Junco hyemalis*) in California found an increased nest success (Bressler et al. 2023). However, a study in Hungary on great tits (*Parus major*) did not find an increase in nest success during the COVID-19 pandemic in urban areas compared to a more natural site (Seress et al. 2021).

Our four target species had low rates of modelled nest survival compared to previous studies in both urban and more natural green spaces. Modelled nest survival rates for American robins have been reported between 1 and 42% in urban areas, depending on the presence of predators (Yen et al. 1996; Malpass et al. 2017), and 43% in more forested, natural sites (Duguay et al. 2001). Gray catbirds are modelled to have 40 to 50% nest survival in both urban and forested habitats (Peak et al. 2004; Piergallini and Yahner 2001; Ryder et al. 2010). Modelled nest survival for yellow warblers in natural forest settings is reported at 30–50% (Humple and Burnett 2010; Briske 1995), but as low as 10% in urban or suburban areas (Strusis-Timmer 2009). Cardinals have lower rates of modelled nest survival in both natural forests (15%) and urban forests (15–25%) (Filliater et al. 1994; Smith-Castro and Rodewald 2010a; Billerman et al. 2022). Our survival rates were much lower than most of these modelled survival rates (Table 4), which could imply these sites are not high-quality habitat for these species. This could be attributed to a variety of factors, including reduced water and food resources leading to poor nutrition (Kark et al. 2007), pollution and contaminants (Isaksson 2015), or higher densities of domestic cats, thought to be the greatest cause of mortality for birds in the United States (Loss et al. 2013).

Contrary to our prediction that nests exposed to higher human activity would experience higher rates of abandonment or predation, we did not find a significant effect of human presence on predicted nest survival. There are a couple of alternative explanations that may explain this finding. Firstly, wildlife in urban spaces may become more tolerant of human presence as time progresses. For example, recent research has found that birds in urban spaces have been less fearful of humans following the COVID-19 pandemic (Diamant et al. 2023). Our target species are also known to be common in urban spaces, meaning that our sampled populations are likely biased towards greater tolerance of human presence and activity. Secondly, recent literature has suggested that human presence is positively related to an increase in bird songs and vocalizations, and authors posited that a habituation process could be the cause, but the human shield hypothesis could also be a driver (Pérez-González et al. 2024; Geffroy et al. 2015; Møller 2012; Valcarcel and Fernández-Juricic 2009). Additionally, bird flight initiation distance (the distance between a human or predator and a bird at which they take flight) is known to be smaller in urban areas compared to suburban areas, further suggesting habituation to humans (Morelli et al. 2022; Møller et al. 2015). Perhaps a reduction in predation and habituation to city life could explain the lack of sensitivity to human disturbance we found.

In contrast to previous studies that suggest nest patch vegetation is influential in nest survival, it was not a significant predictor of nest survival in our study area. Perhaps there are vegetative drivers at play that we did not measure. For example, the amount of suitable nest habitat area within a green space, or the amount of green land cover surrounding each of our sites. Nest survival in ground-nesting species has been shown to increase with an increase in suitable nest habitat for multiple species (Webb et al. 2012; Simonsen and Fontaine 2016), with the amount of green space in the matrix around sites also increasing rates of nest success (Simonsen and Fontaine 2016).

While seasonality was not a significant predictor of nest survival, our seasonality model performed marginally better than the others, indicating that seasonality may influence nest survival in our study area. We found that our target species nesting in urban green spaces are more likely to experience nest failure earlier in the season and most nests are likely to fail before the fifteenth day of the nesting period, regardless of site (Fig. 2). We can also observe that for our four target species, nest failure rates were steepest in the first two weeks of the nesting period, which correspond to nest building, egg laying and early incubation periods (Fig. 2). These periods are when nests are most vulnerable. Birds are more likely to abandon nests before their nestlings hatch – at which point they have invested a lot of energy into their nesting attempt and are less likely to abandon (Verboven and Tinbergen 2002). Furthermore, the incubation period is when nest predation events are known to be most common (Roper and Goldstein 1997; Farnsworth and Simons 1999).

Birds attempting to nest early in the season encounter several challenges that could explain our results. Key hazards to their survival earlier in the nesting season are lower temperatures, decreased food availability, and shorter days (Dunn 2004; Germain et al. 2015; Nager 2006). Producing and incubating eggs is an extremely costly task for the parents, and maintaining an optimal temperature for both their survival and proper embryo development is more difficult in the colder temperatures of the early nesting season (D’Alba et al. 2009; Kim and Monaghan 2006; Nager 2006). Additionally, birds learn from past nesting attempts and can even acquire knowledge on how to best build nests from more experienced adults (Guillette et al. 2016). And lastly, earlier in the nesting season the vegetation is just starting to leaf out, so nests will have less concealment from predators and are easier to find (Ringelman and Skaggs 2019). These factors likely contributed to the lower number of active days experienced by early-season failures and to seasonality being the best predictor of overall nest survival.

A few limitations may have influenced our results. While the study sites varied widely in their access, management, and levels of human activities, they were all representative of larger, more natural North American green spaces. Future research could include areas with even greater human activity, as human influence might become more influential in such areas. There may be a threshold of tolerance or a point at which higher rates of human activity become harmful to nest survival. We also (for logistical reasons) targeted four species that were known to commonly nest in these areas, and as such may be species predisposed to be adaptive to human presence. Other rarer species may indeed be more sensitive to disturbance than the target species of this study, as it was recently shown that 78% of bird species do not tolerate high human-dominant environments (Marjakangas et al. 2024). Future research should also assess the influence of human activity on urban birds in the global south, where differences in urban form and green space design, population densities, and biodiversity may lead to different relationships than those found here (Shackleton et al. 2021).

Within the context of the study’s caveats, generally our findings suggest that human activity and the presence of trails used for human recreation are not negatively impacting the nesting success for our target bird species using urban green spaces. This may tentatively suggest that urban green spaces can provide recreational activity and connection to nature for people without additional negative effects for common nesting birds. Land managers, such as municipal governments, have a great need for such integrated science advice that meets their desire to understand multiple goals of managing urban green spaces. Future research that continues to improve our understanding of synergies and trade-offs between connecting people with natural spaces and human-wildlife interactions is useful in guiding both place-based management and general best practices and policies.

## Supplementary Information

Below is the link to the electronic supplementary material.

ESM 1 (DOCX 168 KB)

## Data Availability

Upon inquiry, data will be provided through a GitHub repository.
