# Supplementary material for: Human recreational activity does not influence open cup avian nest survival in urban green spaces
Source: Urban Ecosyst. 2025 Jan 30;28(2):24. doi: 10.1007/s11252-024-01669-0 (PMC11782460; doi:10.1007/s11252-024-01669-0)
Supplement: Supplementary file 1 — (DOCX 168 KB) [file 11252_2024_1669_MOESM1_ESM.docx]

**Title**: Human Recreational Activity Does Not Influence Open Cup Avian Nest Survival in Urban Greenspaces

**Authors:** Chloe A. Cull^1^, Mackenzie J. Guest^1^, Barbara Frei^1,2^, Carly D. Ziter^1^

**^1^** Department of Biology, Concordia University, Montreal, QC, Canada

**^2^** Environment & Climate Change Canada, Government of Canada, Montreal, QC, Canada

**Corresponding Author**: Chloe A. Cull^1^, chloe.cull@mail.concordia.ca

**Appendix**


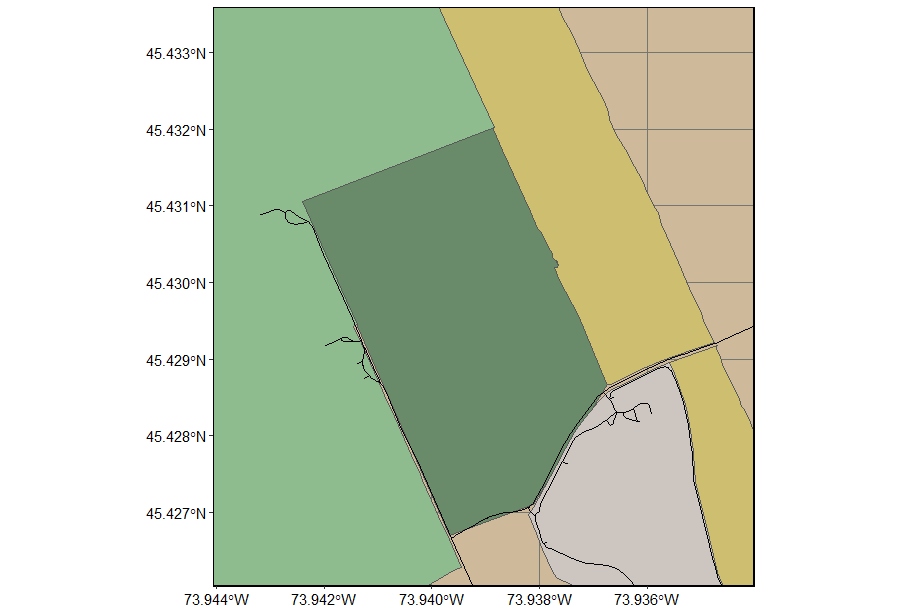

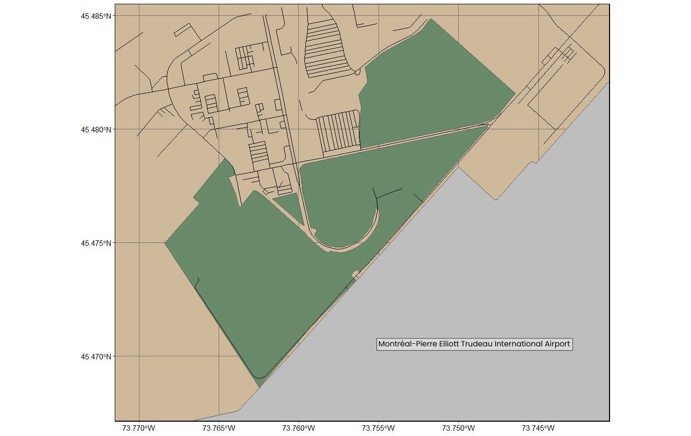

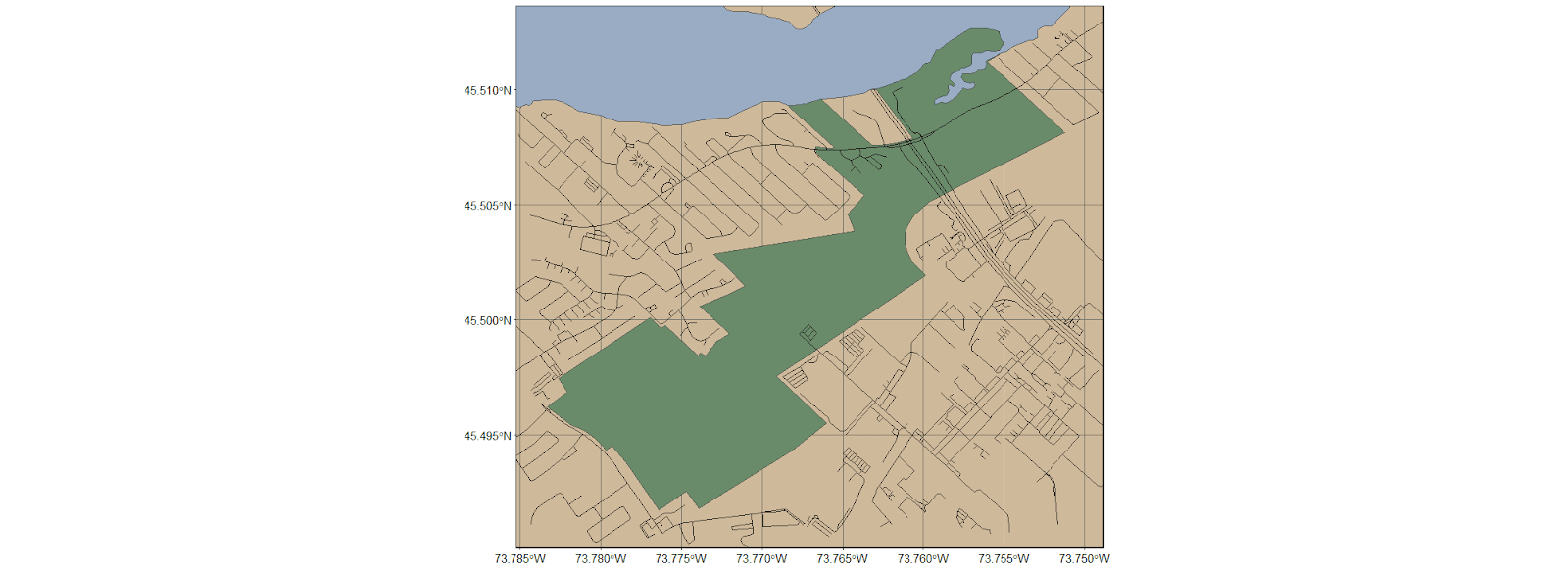


**Appendix A**. Map of study sites on the Island of Montreal, QC. The leftmost depicts the area of Park Bois-de-Liesse (dark green), our high human disturbance site. This site is surrounded by residential, single-family homes. The middle shows the area of the Technoparc (dark green), our intermediate human disturbance site. This site is located between the Montreal-Pierre Elliott Trudeau International Airport (grey) to the south, and an industrial park. The rightmost shows the area of the Stoneycroft Wildlife Area (dark green), our low human disturbance site. This site is adjacent to the Morgan Arboretum (light green) , a forest reserve managed by McGill University. East of the site are agricultural fields (yellow), and to the south is the Ecomuseum Zoo (grey).

**Appendix B.** *Various vegetation survey methods.*

For vegetation complexity considered three layers: understory, midstory, and canopy. We defined understory as any vegetation below hip level, midstory was anything above 6 feet tall, and the canopy layer was consistent with typical definitions of canopy in forestry (Dueser & Shugart Jr. 1978, Canadian Forest Service 2008). We scaled vegetation complexity from 1-3 based on the number of layers present (e.g., a nest patch with an understory and a canopy layer would be coded to have a vegetative complexity of 2).

We took canopy cover photos above each nest that could be reached for a photograph by hand, roughly 1 inch above the nest at a 180° angle. The same person took all photos for consistency. We conducted digital image analysis (Xiong et al. 2019) in ImageJ (Schneider et al. 2012) using an extension called Hemispherical_2.0 to generate canopy cover values (Beckschäfer 2015).

For our vegetative concealment measurements, we used checkerboards of alternating black and white squares. The same observer estimated the number of checkerboard squares that were visible in all four cardinal directions of the nest by standing 3 m from the nest. We took an average concealment value for each nest from these four measurements.

**Appendix C**. All nests found in the study, their location, species, and failure (F) or success (S).

| **Nest Number** | **Site** | **Species** | **Fate** |
| --- | --- | --- | --- |
| 2 | Technoparc | Northern cardinal | F |
| 3 | Bois-de-Liesse | Northern cardinal | F |
| 5 | Technoparc | American robin | F |
| 7 | Technoparc | American robin | F |
| 8 | Technoparc | American robin | F |
| 9 | Technoparc | American robin | F |
| 11 | Bois-de-Liesse | Northern cardinal | F |
| 12 | Technoparc | Northern cardinal | S |
| 13 | Technoparc | American robin | F |
| 15 | Technoparc | American robin | S |
| 16 | Technoparc | Northern cardinal | F |
| 17 | Technoparc | Northern cardinal | F |
| 18 | Technoparc | Northern cardinal | F |
| 20 | Technoparc | American robin | F |
| 21 | Bois-de-Liesse | Northern cardinal | S |
| 22 | Bois-de-Liesse | Northern cardinal | F |
| 24 | Bois-de-Liesse | Northern cardinal | F |
| 25 | Stoneycroft | American robin | S |
| 26 | Stoneycroft | Northern cardinal | F |
| 27 | Technoparc | American robin | F |
| 28 | Technoparc | American robin | F |
| 29 | Technoparc | American robin | F |
| 30 | Technoparc | American robin | F |
| 32 | Bois-de-Liesse | American robin | S |
| 33 | Bois-de-Liesse | American robin | S |
| 34 | Bois-de-Liesse | Northern cardinal | F |
| 36 | Bois-de-Liesse | Northern cardinal | F |
| 37 | Bois-de-Liesse | Northern cardinal | F |
| 38 | Technoparc | American robin | S |
| 39 | Technoparc | American robin | F |
| 42 | Technoparc | American robin | F |
| 43 | Technoparc | American robin | S |
| 44 | Bois-de-Liesse | American robin | F |
| 45 | Bois-de-Liesse | Northern cardinal | F |
| 47 | Stoneycroft | American robin | F |
| 48 | Stoneycroft | Yellow warbler | F |
| 50 | Technoparc | Northern cardinal | F |
| 52 | Bois-de-Liesse | American robin | F |
| 53 | Bois-de-Liesse | Gray catbird | F |
| 54 | Bois-de-Liesse | Northern cardinal | F |
| 55 | Bois-de-Liesse | Gray catbird | F |
| 56 | Bois-de-Liesse | Yellow warbler | F |
| 57 | Bois-de-Liesse | American robin | F |
| 58 | Bois-de-Liesse | Gray catbird | F |
| 60 | Stoneycroft | Gray catbird | F |

**Appendix C**. Continued

| **Nest Number** | **Site** | **Species** | **Fate** |
| --- | --- | --- | --- |
| 61 | Technoparc | American robin | S |
| 62 | Technoparc | Yellow warbler | S |
| 63 | Technoparc | Yellow warbler | S |
| 64 | Technoparc | American robin | F |
| 65 | Technoparc | American robin | S |
| 66 | Technoparc | Yellow warbler | F |
| 67 | Bois-de-Liesse | Northern cardinal | F |
| 68 | Stoneycroft | American robin | F |
| 69 | Technoparc | Yellow warbler | F |
| 70 | Technoparc | Yellow warbler | F |
| 71 | Technoparc | Yellow warbler | F |
| 72 | Technoparc | Yellow warbler | F |
| 73 | Technoparc | Gray catbird | S |
| 74 | Bois-de-Liesse | Gray catbird | F |
| 75 | Stoneycroft | Yellow warbler | F |
| 76 | Stoneycroft | Yellow warbler | S |
| 77 | Technoparc | Gray catbird | S |
| 78 | Technoparc | American robin | F |
| 79 | Technoparc | Yellow warbler | F |
| 81 | Technoparc | American robin | S |
| 82 | Bois-de-Liesse | Yellow warbler | F |
| 83 | Bois-de-Liesse | Gray catbird | F |
| 84 | Bois-de-Liesse | Gray catbird | F |
| 85 | Bois-de-Liesse | Yellow warbler | F |
| 86 | Bois-de-Liesse | Gray catbird | F |
| 87 | Bois-de-Liesse | Yellow warbler | F |
| 88 | Technoparc | Yellow warbler | S |
| 89 | Stoneycroft | Northern cardinal | F |
| 91 | Stoneycroft | Yellow warbler | F |
| 92 | Stoneycroft | Yellow warbler | F |
| 96 | Stoneycroft | Yellow warbler | F |
| 97 | Stoneycroft | Yellow warbler | S |


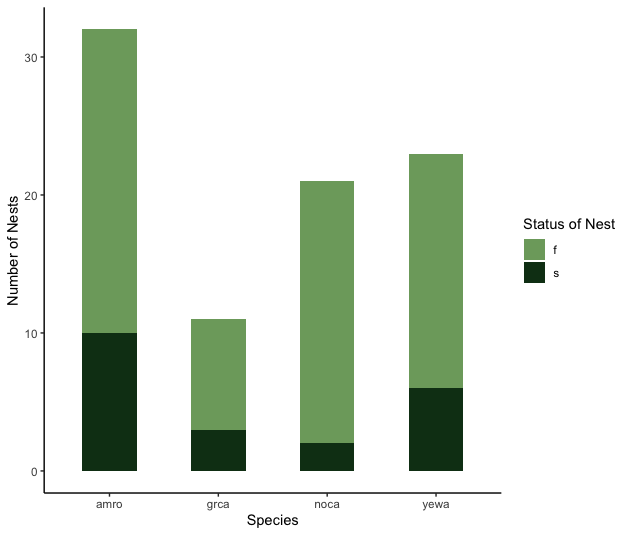


**Appendix D.** The total number of nests found for each of four target species from a nest survival study conducted in the spring and summer of 2023 at Bois-de-Liesse Nature Park, Technoparc’s Parc-des-Sources, and the Stoneycroft Research Area in Montreal. From left to right: American robins (n = 32), gray catbirds (n = 11), Northern cardinals (n = 21), and yellow warblers (n = 23). The pale green at the top of the bars shows the number of nests that failed, and the dark green below indicates the number of successful nests.

**Appendix E.** Candidate models with the two lowest corrected delta AICs (excluding the Null model) predicting nest survival of American robins, gray catbirds, Northern cardinals, and yellow warblers in three urban forests of Montreal are reported, along with their coefficients, exponential coefficients (hazard ratios), standard errors, 95% confidence intervals, z-values and p-values. A hazard ratio > 1 indicates an increase in hazard and a lower probability of survival, while a hazard ratio < 1 indicates an increasing probability of survival.

| **Model and Variables** | **Coefficient** | **Exp** | **SE** | **95% CI** | **Wald z** | **Wald p** |
| --- | --- | --- | --- | --- | --- | --- |
| **Seasonality** |  |  |  |  |  |  |
| Initiation Date | 0.01 | 1.01 | 0.01 | [-0.01, 0.03] | 1.4 | 0.16 |
| **Human Influence** |  |  |  |  |  |  |
| Distance of nest  from trail | 0.05 | 1.05 | 0.22 | [-0.38, 2.46] | 0.22 | 0.83 |
| Mean human  activity | 6.86 | 952.07 | 12.43 | [-17.51, 31.23] | 0.5 | 0.58 |

**Appendix F**. Random Effects Variables for Top Models

For our Cox proportional hazards Seasonality model, the random effect variable of “species” accounted for 0.04 of the observed variance, and the random effect variable of “site” accounted for 0.01 of the observed variance. The random effect variable of species accounted for 0.03 of the variance observed, and the random effect of site accounted for 0.02 of the variance observed for our human influence model.
